# The anatomy of phenotype ontologies: principles, properties and applications

**DOI:** 10.1093/bib/bbx035

**Published:** 2017-04-06

**Authors:** Georgios V Gkoutos, Paul N Schofield, Robert Hoehndorf

**Affiliations:** 1 University of Birmingham Medical School; 2 University of Cambridge; 3 Computer, Electrical and Mathematical Sciences and Engineering Division, King Abdullah University of Science and Technology, King Abdullah University of Science and Technology, Thuwal

**Keywords:** phenotype, ontology, PATO, data integration, Semantic Web

## Abstract

The past decade has seen an explosion in the collection of genotype data in domains as diverse as medicine, ecology, livestock and plant breeding. Along with this comes the challenge of dealing with the related phenotype data, which is not only large but also highly multidimensional. Computational analysis of phenotypes has therefore become critical for our ability to understand the biological meaning of genomic data in the biological sciences. At the heart of computational phenotype analysis are the phenotype ontologies. A large number of these ontologies have been developed across many domains, and we are now at a point where the knowledge captured in the structure of these ontologies can be used for the integration and analysis of large interrelated data sets. The Phenotype And Trait Ontology framework provides a method for formal definitions of phenotypes and associated data sets and has proved to be key to our ability to develop methods for the integration and analysis of phenotype data. Here, we describe the development and products of the ontological approach to phenotype capture, the formal content of phenotype ontologies and how their content can be used computationally.

## Introduction

The distinction between genotype and phenotype was first made by the Danish botanist Wilhelm Johannsen in 1909 [1] and disseminated in a later paper in English in 1911 [2]. Johannsen defined ‘phenotype’ as any observable characteristic of an organism and, in modern terms, ‘genotype’ as the organism’s inherited blueprint, i.e. its genomic information. This definition remains among the best characterizations of what we mean by phenotype, and we understand, as did Johannsen, that an organism’s phenotype arises from the complex interactions between its genotype and its environment.

A major challenge of the past two decades has been the capture of phenotypic information in a way that is amenable to computational analysis. The central problem with describing phenotypes is that they are traditionally described in natural language. Natural language is highly expressive and, given the constraints of a specialist or professional tradition of terminologies and communication styles, extremely effective at capturing and communicating information about phenotypes. However, these traditional methods are subject to ambiguity, modified by context, and the semantic meaning of concepts and terms depends on the background knowledge of domain experts. These challenges make it difficult to use data captured in natural language, computationally [3].

In response to these needs, several disciplines, notably medicine, have developed standardized terminologies over the years to promote a common language of discourse [4]. Such terminologies, while useful, are limited in their computational applicability and face interoperability issues when competing terminologies, or atypical application of terms, result in loss of meaning or translation. This problem has been addressed in recent years through the adoption of structured and semantically formalized ontologies that deal with the issues of both standardization and computability.

The development of bio-ontologies has closely followed the technological developments such as sequencing technologies and gene expression arrays in the biomedical sciences, which resulted in the generation of massive amounts of highly complex data and subsequent analytical challenges. A solution to the problem of data annotation, integration and analysis was developed in the early 2000s with the creation of the Gene Ontology (GO) to describe the attributes of gene products [5].

The GO was the first systematic application of formal ontological principles [6, 7] to the biomedical sciences and facilitated one of the first formal semantic approaches to biomedical data integration. The application of ontologies to data integration has the advantage of allowing capture of relationships between concepts in a domain through formal axioms [7]. The ability of ontologies to capture knowledge through their axioms further permits the use of automated reasoners across them, a property that has become increasingly important in the way ontologies are currently being used [8].

Following the development of GO, there was an efflorescence of ontologies for the capture of phenotypic information, i.e. ontologies of anatomy and of phenotypes mainly aimed at systematically capturing phenotype information from humans and model organisms. The urgent need to develop standards for capturing phenotypic information was recognized as early as 2000 [9]. The human embryological anatomy ontology was developed in 2003 [10] and followed in the same year by the Mammalian Phenotype Ontology (MPO) [11] and the mouse anatomical dictionary [12], and a year later by the Mouse Pathology Ontology (MPATH) [13].

Implementation of these ‘pre-composed’ ontologies, i.e. ontologies where each concept was represented by a single class and its associated label, was largely in the model organism databases such as the Mouse Genome Database [14]. In 2004, the need for a more systematic effort for building phenotype ontologies was recognized [15], resulting in the development of the Phenotype And Trait Ontology (PATO) and the entity–quality (EQ) formalism to describe phenotypes [16, 17]. While the phenotype ontologies provide standards for capturing phenotype information, the PATO framework provides a systematic way to represent phenotypes formally, develop new phenotype ontologies and integrate phenotype ontologies across domains, enabling their comparison as well as, by extension, the comparison of phenotype data associated with them. The unifying PATO framework for describing phenotypes computationally allowed the full potential of ontology-based data integration and analysis to be applied to phenotypes, including semantic similarity measures for determining the phenotypic relatedness of annotated entities [18], semantic search across different ontologies [19] and enrichment over ontologies, for example, to identify biological processes affected by experimental perturbations or natural variation [20].

The systematic application of ontologies for data collection is now becoming the norm for experimental organisms [21, 22], the ecological and evolutionary fields, though more slowly for humans [23]. Here, we review the principles underlying phenotype ontologies and how they give rise to different analytical approaches. We first provide a brief introduction into ontologies and Semantic Web technologies and review the landscape of phenotype and disease ontologies. We then discuss phenotype ontologies that make use of the PATO framework extensively, focusing on the technical and formal details. Following this technical discussion, we highlight applications of phenotype ontologies in biological and biomedical data analysis and provide conclusions and an outlook for phenotype ontologies. 

## Ontologies and the Semantic Web

Most modern ontologies in biology and biomedicine use the Web Ontology Language (OWL) [24] to express their content. OWL is a formal language based on description logics [25], and uses several profiles that correspond to different language subsets [26]. OWL makes a basic distinction between classes and instances. A class is an OWL entity that can be instantiated and usually classes correspond to general kinds of entities. Examples of classes are ‘Arm’ (a kind of material structure), ‘Drinking’ (a kind of process) or ‘Color’ (a kind of quality). Instances, on the other hand, cannot have instances themselves and correspond to the things that are found within a domain of interest. Examples of instances are ‘my right arm’, the ‘drinking process mouse A was involved in at a particular period’ or the ‘color of my skin’.

Most ontologies contain no instances and specify the classes within a domain of interest through the use of axioms [8]. An axiom is a statement assumed to be true within a domain of knowledge. Some of the simplest axioms assert that one class is a subclass of another, conveying the information that all instances of the former are also instances of the latter. More complex axioms can be formed through the use of quantifiers and relations (object properties and datatype properties in OWL) that hold between instances.

Ontology axioms can be used together with automated reasoning to reveal implied, albeit not explicitly stated, knowledge. For example, from the two axioms that A SubClassOf: B and B SubClassOf: C, it can be inferred that A SubClassOf: C. Reasoning over OWL ontologies can be highly complex and is, in general, exponential in the size of the OWL knowledge base [24]. However, some fragments of OWL have been defined for which inferences can be performed in polynomial time [26]. In biological, biomedical and medical ontologies, the OWL 2 EL profile is widely used, as it supports many of the axioms and operations useful to express biological knowledge while maintaining polynomial time complexity so that it can be used together with large ontologies [27]. In particular, OWL 2 EL supports subclass axioms (such as red SubClassOf: color), equivalent class axioms (such as ’high blood pressure’ EquivalentTo: hypertension) and disjointness axioms (such as red DisjointWith: blue), as well as class constructors including existential restrictions and class intersection (such as ’part of’ some liver and ’part of’ some ’cardiovascular system’). OWL 2 EL further allows the use of transitive and reflexive properties, and supports combining multiple properties through properties chains. The specification of OWL profiles provides a comprehensive reference [26].

## Landscape of phenotype ontologies

Three major strategies for the capture of phenotype data dominate the ontology landscape. Most widely used are ontologies containing pre-composed classes describing ‘abnormal’ phenotypes aiming either to completely describe the abnormal phenome of an organism or specific phenotypic domains such as disease or behavior. Examples are the MPO [11], the Human Phenotype Ontology (HPO) [28] or the Fission Yeast Phenotype Ontology (FYPO) [29], all of which aim to capture aberrations related to the entire phenome of an organism. This approach assumes the presence of a reference organism or strain, and ‘abnormal’ phenotypes denote a deviation from this norm. In contrast, the trait ontologies such as the Wheat Trait Ontology (part of the Crop Ontology) [30, 31] capture individual traits and their units of measurement [32] when they represent continuous variables, without including any ‘normal’ and ‘abnormal’ phenotypes within the ontology. The third approach is to record an entity (or entities) involved in a particular phenotypic manifestation and the manner that it has been affected. This approach takes the entity from a selection of ontologies (most often, ontologies of anatomy and physiology) and the quality from PATO [16]. An example of this approach is the Zebrafish Information Network (ZFIN) [33, 34] that uses this ‘post-composed’ approach to phenotype descriptions, using, among others, the GO [5] and the Zebrafish Anatomy Ontology [35] to refer to the entities.

Table 1 presents a survey of pre-composed phenotype ontologies and trait ontologies for humans, several model organisms, plants and microorganisms gathered from Bioportal [73], Aber-OWL [74] and the Ontology Lookup Service [75]. The list provided in Table 1 is not intended to be comprehensive and contains ontologies, which are widely used across many projects and databases as well as ontologies restricted to specific applications.

**Table 1:** Landscape of phenotype ontologies

Domain	Ontology	# Classes	Used by
Human and biomedical	DO [36]	11 663	DisGeNet [37]
	HPO [38]	15 381	HPO Database [39] and GWAS Central [40]
	International Classification of Disease version 10, Clinical Modification (ICD10CM) [41]	92 168	Various EHR systems
	ICD9CM [42]	22 533	Various EHR systems
	Medical Subject Headings Thesaurus [43]	261 990	Comparative Toxicogenomic Database [44]
	UMLS [43]		SIDER [45] and DisGeNet [37]
	NCI Thesaurus [46]	118 941	NCI, NIH multiple projects
	Ontology of Adverse Events [47]	5514	
	Orphanet Rare Disease Ontology [48]	13 105	Orphanet [49]
	Read Codes Clinical Terminology version 3 [50]	140 065	UK General Practice
	SNOMED-CT [51]	324 129	Various EHR systems
Animal model organism	Dictyostelium Phenotype Ontology [52]	1058	DictyBase [52]
	DPO [53]	506	FlyBase [54]
	MPATH [13]	889	PathBase [55], RGD [56] and MGI [14]
	MPO [22]	30 316	MGI [14], RGD [56]
	Worm Phenotype Ontology (WBPhenotype) [57]	2435	WormBase [58]
Plants and fungi	Ascomycete Phenotype Ontology	619	Saccharomyces Genome Database [59]
	Flora Phenotype Ontology [60]	28 430	African Plants Database [61]
	FYPO [29]	9870	PomBase [62]
	TO [63]	1433	iPlant Collaborative Databases [64] and Planteome [65]
	Solanaceae PATO [30, 31]	397	Sol Genomics Network [66]
	Thesaurus Of Plant traits [67]	950	TRY Database [68]
Cell	Cell Microscopical Phenotype Ontology [69]	813	Cellular Phenotype Database [70]
	Ontology for Microbial Phenotypes (OMP) [71]	1120	Microbialphenotypesȯrg [72]

### Ontologies for the medical sciences

The terminologies for collecting information on human diseases have a long history, with the key terminologies being the Unified Medical Language System (UMLS) [43], the Systematized Nomenclature of Medicine-Clinical Terms (SNOMED CT) [51], the International Classification of Diseases (ICD) codes [41] and the UK Read codes [50]. While these terminologies are hierarchically structured, they were not originally intended to be used as ontologies and followed the pattern of the Simple Knowledge Organization System (SKOS) [76]. In recent years, however, they have been rendered into formal ontological formats, which can be used to support some operations commonly performed on ontologies. The UMLS represents one of the most useful resources for biomedical semantics, partly because of its size and comprehensive coverage, but mainly because of the extensive mapping to other ontologies, allowing the UMLS to bridge terminological and structural gaps between different ontologies. Many databases use UMLS along with other ontologies for coding information about disease and phenotypes, such as DisGeNet [37] or the Comparative Toxicogenomics Database [44].

More recently, the HPO [38] was introduced to specifically characterize phenotypes in humans and represent human diseases as a combination of phenotypes. Applied to patients, the HPO enables highly granular, ‘deep’, phenotyping [77], which has been shown to be highly successful in increasing the accuracy of diagnosis, facilitating patient population stratification and identifying novel candidate genes for genetic disorders [18, 78]. Consequently, the HPO is now applied by a large number of projects and consortia aimed at characterizing patients and understanding molecular mechanisms underlying their diseases, including the UK 100 000 Genomes project [79]. Furthermore, journals have started to require the submission of HPO class-based phenotype descriptions with manuscripts that characterize patients or diseases [80] so that information about observed phenotypes can be reused more widely. HPO is also used for indexing and annotating information in human genetics databases, such as the Online Mendelian Inheritance in Man [81] and Orphanet [49] databases, GWAS Central [40] and ClinVar [82] as well as clinical electronic health record (EHR) systems.

A similar move toward more formalization and precision can be observed in terminologies intended to capture diseases, where the Disease Ontology (DO) [36, 83] aims to capture all human diseases. DO provides mappings to traditional clinical terminologies such as SNOMED CT or International Classification of Diseases version 9, Clinical Modification (ICD9CM), and some databases fully characterize diseases in DO with phenotypes from ontologies such as HPO [84].

Medical terminologies have traditionally focused on classifying the terminology used to characterize the state of a patient, and they often contain terms that may both refer to a phenotype or a disease. While the distinctions between phenotypes and diseases are not always obvious, there is now a strong tendency in the biomedical ontology community to consider diseases as a priori distinct from phenotypes [85–87]; a disease would usually consist of multiple different phenotypes. There are many challenging cases, such as hypotrichosis, which may be considered a phenotype if occurring with other signs or symptoms or as a disease in itself if isolated (e.g. as generalized hypotrichosis).

The overlap between disease and phenotype ontologies allows ontology mapping techniques to be applied to identify overlapping or equivalent classes in different ontologies [48, 88, 89]. Inter-ontology mapping not only enables ontology interoperability, but also permits the integration of large clinical data sets. The coding of drug side effects and indications to UMLS, SNOMED CT, ICD9CM and the Medical Dictionary for Regulatory Activities (MedDRA) adverse event terminology further permit the integration of drug, pathway and disease data from databases such as PharmGKB [90], Drugbank [91] and SIDER [45] with other resources, and have been used to predict both novel drug targets and indications [92–95]. In these approaches, phenotype ontologies are used to combine data sets both to increase sample size and connect phenotypes observed under different circumstances, such as drug effects and mutant model organism phenotypes.

### Model organism phenotype ontologies

The model organism databases, such as the Mouse Genome Informatics (MGI) database [14], provide ontology-coded genotype/phenotype information for natural and induced mutations, and they were the first resources to use formal ontologies for phenotype coding. In these databases, most phenotype information is captured by literature curation or by direct coding from large-scale high-throughput projects such as the International Mouse Phenotype Consortium [96, 97] and industry data sets from Lexicon and Deltagen.

Computational integration of phenotype data between species has been made possible through a combination of lexical matching of class labels, PATO-based standardization of phenotype ontologies and cross-species anatomy and physiology ontologies such as Uberon [98] and GO [5]. Together with reasoning over multiple ontologies (as described in detail below), this has allowed model organism phenotypes to directly contribute to translational research, for example, predicting the causative genes of rare human diseases [99], identification of the contribution of component genes in contiguous gene syndromes [100] and, more recently, prioritizing causative variants from undiagnosed clinical exomes [101]. Here, integration of phenotype ontologies is used to make phenotypes in different species comparable, while measures of semantic similarity are used to associate the perturbations (such as targeted mutation) underlying the phenotypes and generate hypotheses about the mechanisms leading to a phenotype when the underlying causes are unknown.

### Biodiversity and ecology

Systematic collection of phenotype data as part of the characterization of natural environments has always been an integral part of ecology and biodiversity studies. The increasing scale and complexity of data collection, the establishment of genomic observatories and new methods of large-scale analysis have stimulated the development of tools for biodiversity informatics [102, 103] among which the plant and fungi ontologies listed in Table 1 represent the major ontological tools now in use. Environment is a critical aspect of phenotype interpretation and particularly relevant in a biodiversity and ecological context where environments are not standardized, and the Environment Ontology (ENVO) [104] provides a tool to capture a broad range of environmental data [105]. There are also more specialized ontologies, such as the Plant ENVO [63] for plant environmental conditions. Notably, in the plant sciences (including biodiversity and ecology, but also spanning agriculture and plant model organisms), large collaborative efforts, in particular the Planteome project [65], the Crop ontology project [30, 31] and the iPlant Collaborative [64], have undertaken to unify and standardize the different vocabularies, ontologies and databases.

### Evolutionary biology

The collection of phenotypic characteristics for the study of evolution using natural language has been established for a considerable time [106]. More recently, phylogenetic systematics has formalized the collection of comparative data across taxa; yet, much of the collected phenotypic information is in publications inaccessible to computational analysis, and impossible to integrate with the information in genetic and phenotypic databases. The difficulties in analyzing and integrating such data have been successfully addressed using anatomy and phenotype ontologies underpinned by the PATO framework [107]. This approach has been successfully applied in many projects, most notably in the Phenoscape project [108, 109] where a knowledge base constructed from ontology-based literature mining has been used to predict the genes responsible for phenotypic differences between related species, both extant and extinct. The hypothesis underlying these studies is that observed phenotypic differences between two species resulting from evolutionary change in a single gene or pathway can be compared with differences between a normal and mutant model organism, and if the observed differences are similar, the evolutionary change likely happened in the same gene or pathway that has been altered in the mutant model organism.

### Agricultural and livestock ontologies

Several ontologies have been created specifically in support of domestic animal production, which cover specific phenotypic traits of multiple food species. In the Animal Trait Ontology for Livestock (ATOL) [110], for example, classes refer to traits and not abnormal phenotypes and are closely related to industry-standard measurement techniques facilitating the capture of quantitative and discrete data using the EQ formalism. The ATOL is designed to work with the environment ontology for livestock, providing a matched pair of resources that can capture all the parameters needed to interpret phenotypic findings.

### Application ontologies containing phenotypic concepts

An application ontology is an ontology generally derived for a specific use or application [111], often to model a broad range of aspects of a specific domain, or sometimes to mirror the content of a database containing data on a specific domain. We do not include application ontologies in Table 1 because of space constraints and the large number of application ontologies containing phenotypic classes. Many of the major application ontologies follow the Open Biological and Biomedical Ontologies (OBO) Foundry principles [112] and reuse classes from phenotype ontologies using the Minimum Information to Reference an External Ontology Term approach [113]. Prominent examples of these application ontologies include the Experimental Factor Ontology [114], used across the European Bioinformatics Institute (EBI) databases; the Ontology of General Medical Science [87], intended to unify clinical ontologies used in EHR systems; the Neuro Behavior Ontology [115], used in axioms of several phenotype ontologies and for annotation by resources such as the Rat Genome Database (RGD) [56]; and the Infectious DO [116], used as a foundation to build several disease-specific ontologies.

## PATO-based phenotype ontologies

**Figure 1:**
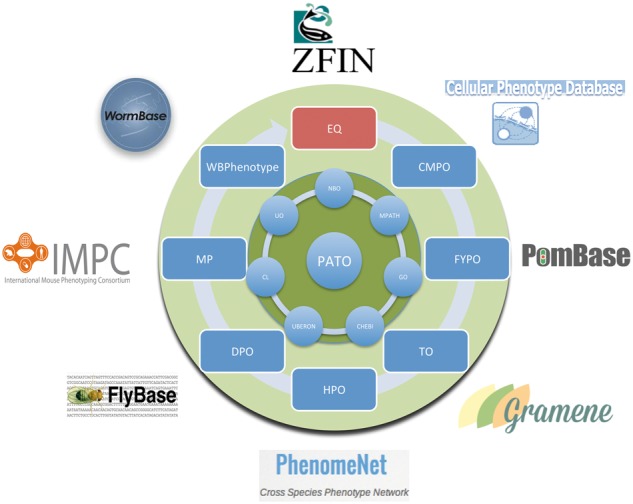
A schematic representation of the PATO framework. Quality classes from PATO are combined with entity classes from multiple ontologies (such as GO or Uberon) to provide formal definitions for species (and sometimes domain specific) phenotype ontologies. Examples of such ontologies are depicted in the outer ring and include the HPO [118], MPO [119], Cellular Phenotype Ontology (CPO) [120], Drosophila phenotype ontology (DPO) [53], Plant Trait Ontology (TO) [117] FYPO [121] and Wormbase Phenotype Ontology [57]. This provides an interoperability layer between these ontologies and facilitates the integration of the data annotated to them in the species-specific databases around the outside edge.

The PATO [16] was built as a framework to unify phenotype descriptions in biology, render them interoperable and make them amenable to automated reasoning and processing (see Figure 1 for an overview). The PATO framework [16] is not only an ontology but also provides a uniform way to express phenotype statements, the EQ method [117]. According to the EQ method, a phenotype is described by referring to an ‘entity’, either an anatomical structure or a biological process or function, and characterizing this ‘entity’ with a ‘quality’ that captures the properties or attributes of the entity. When using the EQ method to describe phenotypes, the entity will usually refer to a class from either an anatomical ontology or an ontology of processes and functions such as the GO [5], while the quality is taken from the PATO. For example, to describe the trait ‘heart morphology’ using the EQ method, the class ‘heart’ (from an anatomy ontology) is used as entity and the class ‘morphology’ (from PATO) is used as quality. To describe ‘heart hypertrophy’, the same class ‘heart’ is used as entity and the quality ‘hypertrophic’ from PATO. The class ‘hypertrophic’ in PATO is a subclass of ‘morphology’ and should result in ‘heart hypertrophic’ being a subclass of ‘heart morphology’.

A distinction can be made between ‘morphology’, which is an attribute, and ‘hypertrophic’, which is a value of the attribute, and PATO contains this distinction as well as others. The PATO [16] has grown over time and evolved to incorporate distinctions between attributes and their values, qualities and quantities, normal and abnormal qualities, increased and decreased values and unary and n -ary qualities. The rich information in PATO has also led to a complexity that can make the ontology and EQ framework challenging to use, and here we discuss all features of PATO in detail and aim to make them more accessible.

The distinction between attributes and their values is made in PATO through a combination of formal subclass axioms and OBO ‘slims’ [122]. A slim of an ontology is a subset of the ontology consisting of classes and axioms that are useful for a particular purpose, and they are expressed by an annotation property. Examples of attributes in PATO include ‘morphology’, ‘color’ or ‘shape’, while examples of values include ‘hypertrophic’, ‘red’ or ‘round’. Attributes and values are distinguished in PATO through the use of the ‘attribute’ and ‘value’ slims. In the OBO format [123] of PATO, these are expressed as ‘slims’ [122], i.e. application-specific subsets of PATO; in the OWL format of PATO, the distinction is made through the use of annotation properties attributed to the PATO class. Attributes can only have certain values, depending on the value space underlying an attribute [124]. For example, ‘hypertrophic’ can be a value of ‘morphology’ but not of ‘color’. In PATO, possible values of an attribute are subclasses of the attribute class, i.e. PATO classes expressing attributes have their potential values as subclasses. Specifically, given a PATO class expressing a value (such as ‘hypertrophic’), the corresponding attribute is the most specific superclass that falls in the attribute subset of PATO.

Among the classes expressing attributes in PATO [16], a further distinction is made between scalar attributes and non-scalar attributes. Scalar attributes have values that can be partially ordered and have magnitudes, and include attributes such as ‘speed’ or ‘size’. Non-scalar attributes (such as ‘color’ or ‘shape’) have values that are qualitative and do not give rise to a natural ordering. Only scalar attributes can be increased or decreased while it makes no sense to state that a non-scalar attribute is increased or decreased. However, both scalar and non-scalar attributes may have values that are opposites of each other. For scalar values, PATO contains axioms that specify that a certain value of an attribute is ‘increased in magnitude relative to’ or ‘decreased in magnitude relative to’ a reference value. PATO further includes axioms that relate values of attributes to their opposite values, using the ‘opposite of’ relation. For example, the quality ‘rough’ (PATO:0000700) is declared as an ‘opposite’ of the quality ‘smooth’ (PATO:0000701). These axioms can be used to identify phenotype statement that express opposite directionalities. The distinction between scalar and non-scalar attributes is not always clear, as some attributes may be considered to be both scalar and non-scalar, depending on context. Examples include color, which can either be scalar, when expressed as wavelengths, or non-scalar, when expressed using qualitative values such as ‘red’ or ‘yellow’. The scalar versus non-scalar distinction in PATO should therefore be considered a guideline, not an absolute truth.

Another subset of PATO [16] distinguishes between unary and relational qualities. A unary quality is a quality of a single entity, while relational qualities are qualities of multiple entities. Examples of relational qualities include ‘anterior to’ (PATO:0001632) or ‘sensitivity toward’ (PATO:0000085), which are qualities of two entities. In PATO, unary qualities are distinguished from relational qualities through a slim (or an annotation property in the OWL version of PATO) that tags some attributes and values as relational if they are qualities of more than one entity. Relational qualities in PATO are expressed as qualities of one of their bearers and are related using the ‘toward’ relation to their second (and third, fourth, etc.) arguments. For example, the ‘sensitivity toward oxygen’ of a microorganism would be expressed as a quality ‘sensitivity toward’, which is the quality of the microorganism and is directed ‘toward’ some oxygen (towardsome oxygen).

### Direct and comparative phenotype descriptions

Phenotype ontologies can be broadly distinguished in two main classes based on whether they express ‘direct’ phenotype observations or ‘comparative’ observations. We consider direct phenotypes to be raw observations of a single organism, without a reference to another organism for comparison. Direct phenotypes are often collected in a biodiversity and evolutionary context, and they may form the basis of GWAS or PheWAS studies [40, 125]. For example, character matrices will represent phenotypes of individual organisms or species, without comparing them a priori to other entities. Similarly, in a biodiversity context, such as floras or monographs focusing on the organisms within a region, or characterizing a family of related organisms, phenotype descriptions are those of individual organisms or species, without including a comparison [60].

**Figure 2:**
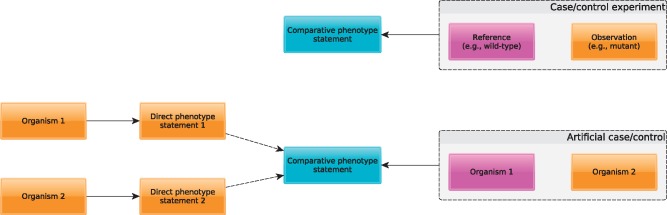
Direct and comparative phenotype statements, and conversions between them. The upper part of the figure shows how phenotype statements are made in case/control experiments, in which the comparative phenotype statement expressed the difference of an observation to an explicitly or implicitly specified control. In the bottom figure, direct observations are made to two organisms, Organism 1 and Organism 2, and the comparative phenotype statements are derived by designating one of the two organisms as control and computing the differences between the other organism and the control.

On the other hand, comparative phenotype statements characterize the outcomes of an experiment, or the observed differences from an explicit or implicit reference state. For example, comparative phenotype statements in a model organism context are statements denoting phenotypic differences resulting from mutagenesis experiments. In the comparative case, a ‘control’ is defined, usually a wild-type of an organism, and a ‘case’ studied, often a mutant or an organism that has undergone a specific procedure such as drug treatment or other environmental effect. The comparative case is illustrated in the top of Figure 2.

Direct and comparative phenotype statements are clearly distinct: while a direct phenotype statement conveys information about exactly one organism, a comparative phenotype statement conveys information about at least two different organisms, case and control. Two direct phenotype statements can give rise to a comparative phenotype statement if the direct phenotype statements are ‘comparable’. Two phenotype statements are comparable if and only if they involve the same kind of entity and ‘comparable qualities’. PATO qualities are comparable if and only if they are values of the same attribute. The attributes to which a PATO quality belongs can be identified in PATO by identifying the most specific superclass of a quality that is tagged as belonging to the ‘attribute’ subset. Given two comparable direct phenotype statements $P_{1}$ (about organism $O_{1}$) and $P_{2}$ (about organism $O_{2}$), with A being the attribute underlying $P_{1}$ and $P_{2}$ and E the entity, and selecting $O_{1}$ as reference, the comparative statement assigned to $O_{2}$ (relative to $O_{1}$) is abnormal/divergent E A (such as ‘abnormal flower color’ for entity ‘flower’ and Attribute ‘color’). For example, when comparing flower red and flower yellow, and using either the first or second as reference, the comparative phenotype is abnormal/divergent flower color because the entity (flower) is identical in both statements and the qualities are comparable (in virtue of both being values of ‘color’) yet different. If $P_{1}$ and $P_{2}$ are based on ‘scalar qualities for which values can be compared quantitatively, more detailed comparative phenotype statements can be inferred involving ‘increased’ or decreased’ values. Figure 2 illustrates the relation between direct and comparative statements.

Identification of comparative phenotypes can be extended from the simple case of two comparable phenotype statements to the case in which sets of phenotypes are observed and a set of comparative phenotypes is generated from these observations. Given two sets of phenotype statements $S_{1}$ and $S_{2}$, the first step is to identify the set of pairs of phenotype statements (x,y) such that $x\in S_{1}$ and $y\in S_{2}$ and x and y are comparable. Assuming $S_{1}$ is selected as control and $S_{2}$ as case, then, for each pair of phenotype statements (x,y), a comparative phenotype can be generated following the procedure above to generate a set of comparative statements. These sets can also directly be recorded in a database, and model organism databases such as MGI and ZFIN, but also human phenotype databases such as the HPO database, record phenotypes in this form.

When collecting phenotype information from mouse populations of the same genotype, the International Mouse Phenotyping Consortium (IMPC) uses statistical analyses to determine significant phenodeviance from the inbred background strain and then codes to the abnormal phenotype class, such that the categorical annotation is to the strain and not to the individual. Each assay is assigned to a set of classes in the MPO that might be called, and this is done automatically in most cases. The PATO on its own does not contain mechanisms to record additional provenance information and evidence about how a phenotype statement was derived. However, the PATO framework [16] originally incorporated an assay ontology, which could have been used to formally characterize the assays and statistical methods that were used to derive a phenotype statement, such as the complex set of assays and statistical methods used by the IMPC [126]. While a dedicated and comprehensive assay ontology has not yet been established, the Ontology of Biomedical Investigations [127] and Evidence Code Ontology [128] can be used for this purpose.

### Interoperability with anatomy and physiology ontologies

The organization of classes in phenotype ontologies usually follows the structure of anatomy and physiology of the organism for which phenotypes are recorded. Many phenotype ontologies were built manually by domain experts, and these ontologies follow the structure of anatomy or physiology ontologies implicitly. However, some phenotype ontologies also make explicit use of the knowledge in anatomy or physiology ontologies, and automatically generate a taxonomic structure through automated reasoning.

Phenotype ontologies use the structure of anatomy and physiology ontologies in several ways:

Taxonomic relations: If C is a subclass of D in an anatomy or physiology ontology, then ‘C phenotype’ is a subclass of ‘D phenotype’ in the corresponding phenotype ontology. For example, ‘T-cell apoptosis’ is a subclass of ‘apoptosis’ in GO, and ‘abnormal T-cell apoptosis’ is a subclass of ‘abnormal apoptosis’ in the MPO.

Anatomical and physiological parthood: If C is a subclass of ‘part of some D’ in an anatomy of physiology ontology, then ‘C phenotype’ is a subclass of ‘D phenotype’ in the corresponding phenotype ontology. For example, ‘left ventricle’ is a subclass of ‘part of some heart’ in the Foundational Model of Anatomy (FMA), and ‘abnormal left ventricle’ is a subclass of ‘abnormal heart’ in the HPO.

Anatomical function: If C is a subclass of ‘function of some D’, then ‘C phenotype’ is a subclass of ‘D phenotype’. Axioms using the ‘function of’ relation are rarely included in current ontologies such as GO, and this rule can also be used in reverse to infer from phenotype ontologies the functions of anatomical entities [129]: if ‘C phenotype’ has manually been asserted to be a subclass of ‘D phenotype’, and C is a class of functions and D a class of material entities, then C should be a subclass of ‘function of some D’. For example, ‘Arrhythmia’ (HP:0011675), based on the entity ‘heart contraction’ (GO:0060047) in the HPO, is asserted to be a subclass of ‘abnormality of cardiovascular system physiology’ (HP:0011025), based on the anatomical entity ‘cardiovascular system’, thereby implying that one of the functions of the cardiovascular system is ‘heart contraction’. Similarly, in the MPO, ‘impaired hearing’ (MP:0006325, based on the entity ‘sensory perception of sound’) is a subclass of ‘abnormal ear physiology’ (MP:0003878, based on the entity ‘ear’), implying that one of the functions of the ‘ear’ is ‘sensory perception of sound’.

These general rules can be used to automatically generate a PATO-based backbone taxonomic structure of a phenotype ontology. For example, such an automated approach has been applied in generating prototypes of the Flora Phenotype Ontology [60] or the Cellular Phenotype Ontology [120]. Additionally, for manually generated phenotype ontologies, formal PATO-based ontology definitions can be created for some or all classes and the background knowledge in anatomy and physiology ontologies can be used to improve the ontology quality.

When these universal rules for structuring PATO-based classes of phenotypes are combined with ontologies representing entities that cross multiple species, they also allow the integration of phenotypes between different species. In particular, when these structuring rules are applied in phenotype ontologies for different species, and the entity class is either selected from an ontology such as Uberon [98] or the GO [5], both of which cover multiple species, or can be mapped to such an ontology, phenotypes can be integrated, and compared, across different species. Such an approach was firstly applied systematically in the PhenomeNET system [130] and later gave rise to approaches such as PhenoDigm [78] and the Monarch Initiative [19].

### PATO-based axiom patterns

Using reasoning over phenotype ontologies necessitates that axioms clearly specify the intended meaning of a class and follow a pattern that is amenable to the desired inferences. The core part of a phenotype description is the ‘entity’ and the ‘quality’ on which a phenotype description is based [16]. There are two main ways in which a phenotype class in an ontology can be specified, using either the ‘quality’ as the primary mode of classification and the ‘entity’ as a modifier [117], or using the ‘entity’ as primary mode of distinction and the ‘quality’ as modifier [131].

To define phenotype class, given an entity E and a quality Q, the basic axiom pattern is P EquivalentTo: ’has part’ some (Q and ’inheres in’ some E). This pattern can be extended to capture more complex phenotypes (i.e. those involving additional modifiers) by further restricting the entity E or the quality Q classes. This pattern is applied in some phenotype ontologies such as the HPO and MPO; yet, it does not on its own generate the backbone taxonomic structure used in phenotype ontologies (which primarily follows anatomy and physiology). To generate the backbone taxonomic structure based on parthood relations in anatomy or physiology ontologies, a relation ‘inheres in part of’ is introduced and used to define phenotype classes for which inheritance over parthood is desirable (such as ‘abnormality of head’ and ‘abnormality of face’). Additionally, for the ‘inheres in part of’ relation, it is asserted that an ‘inheres in’ relation followed by a ‘part of’ relation implies the presence of an ‘inheres in part of’ relation: ’inheres in’ o ’part of’ SubPropertyOf: ’inheres in part of’ (where ‘o’ is the operator to concatenate two object properties in Manchester OWL syntax [132]). With these axioms in place, use of the ‘part of’ relation in anatomy or physiology ontologies can generate new inferred taxonomic relations in phenotype ontologies. For example, the axioms constraining the classes ‘Abnormality of head’ (HP:0000234) and ‘Abnormality of face’ (HP:0000271) in the HP are as follows:

’Abnormality of the face’ EquivalentTo: ’has part’ some

(quality and ’inheres in part of’ some face)

and

’Abnormality of the head’ EquivalentTo: ’has part’ some

(quality and ’inheres in part of’ some head)

Together with the axiom in the anatomy ontology Uberon (from which the classes ‘head’ and ‘face’ are reused) face SubClassOf: ‘partof’some head, it is possible to infer that ‘Abnormality of face’ is a subclass of ‘Abnormality of head’.

An alternative approach is to use the ‘entity’ as primary mode of distinguishing phenotypes and qualities as modifiers [131]. The basic axiom pattern in this approach is to define a phenotype class P based on entity E and quality Q as P EquivalentTo: has-part some (E and has-quality some Q). This pattern results in phenotype classes affecting the same entity to be grouped together, e.g. all ‘Heart’ phenotypes will become sibling classes. To generate a backbone taxonomic structure using the background knowledge in anatomy and physiology ontologies about parthood relations, the axiom pattern has to be modified to replace E with ’part of’ some E: P EquivalentTo: ’has part’ some (’part of’ some E) and ’has quality’ some Q.

Both axiom patterns can be refined by further constraining either the entity or quality within them. Constraints on the entity could be to specify its physical location or development stage, such as in ‘blood located in the left ventricle’ used to define ‘stroke volume’. Qualities can also be further constrained. For example, such constraints are used in relational qualities such as ‘decreased susceptibility to viral infection’ (MP:0002410) based on the quality ‘decreased sensitivity toward’ (PATO:0001550) and ‘defense response to virus’ (GO:0051607).

One controversial topic in providing axioms for classes in phenotype ontologies has traditionally been the treatment of absence [133, 134]. Some discussions center around the ontological status of ‘absent’ entities where it is questioned whether ‘absent’ entities (such as an ‘absent heart’) can exist and have a place in an ontology. One solution to this problem is to treat ‘absent’ entities as linguistic short forms for ‘absence of’ phenotypes. For example, despite its linguistic implications, an ‘absent heart’ can be considered as an ‘absence of a heart’ in an organism, or, more precisely, an organism without a heart as part [131]. We focus here on the functional requirements for classes representing absences. A desirable feature of classes representing absence of anatomical or physiological entities is that the absence of all entities E implies the absence of all parts of E. For example, if an organism has an ‘absence of a heart’ phenotype, it will also have an ‘absence of left ventricle’, ‘absence of aortic arch’, etc. These implications are expressed as subclass axioms in OWL so that ‘absence of a heart’ becomes a subclass of ‘absence of left ventricle’ and ‘absence of aortic arch’. To achieve this classification through automated reasoning, axiom patterns that involve negation need to be used [131]. In particular, an ‘absence of *E*’ can be defined as equivalent to not ’has part’ some (’part of’ some E) when the relation ‘part of’ is considered as reflexive and transitive. For example, with ‘left ventricle’ being a part of the heart, the phenotypes ‘absence of heart’ and ‘absence of left ventricle’ can be defined as equivalent to not ’has part’ some (’part of’ some heart) and not ’has part’ some (’part of’ some ’left ventricle’), respectively. Use of an automated reasoner will then generate the desired inference that ‘absence of heart’ is a subclass of ‘absence of left ventricle’. However, the disadvantage of these axioms is the use of negation, which requires use of an expressive fragment of OWL for which polynomial time automated reasoning cannot be guaranteed. Consequently, axioms of this type are not yet widely applied in phenotype ontologies.

Another controversial topic in phenotype ontologies is the ontological status of phenotypes [112, 87, 135]. This question is mainly relevant when considering how to align a phenotype ontology to an upper-level ontology such as the Basic Formal Ontology [111]. In most cases, phenotypes are considered as specific kinds of qualities, entities that are existentially dependent on a single kind of entity (the quality bearer) throughout their life. However, neither of the patterns we discussed here is amenable to such an interpretation because of the use of the ‘has part’ relation in the beginning of each axiom pattern, which is not usually considered to be applicable to qualities [124, 136, 137]. However, phenotype classes, as they are currently used in phenotype ontologies, can be considered to be either material entities (i.e. whole organisms) that have certain characteristics (having parts with certain qualities) or they can equivalently be considered as qualities. The latter choice necessitates a slight alteration of the axiom patterns by prefixing all of them with ‘inheres in’ some. For example, instead of defining a phenotype P as before, the pattern P EquivalentTo: ’inheres in’ some(’has part’ some (E and has-quality some Q)) can be used. As the ‘inheres in’ relation is functional (i.e. a quality can only inhere in exactly one entity), the original axiom pattern and this modified axiom pattern result in exactly the same inferences and are therefore functionally equivalent. The only difference is that with the original axiom pattern, phenotypes are material entities (i.e. whole organisms), while they are qualities with the modified axiom pattern.

## Ontology-based analysis of phenotypes

Phenotype ontologies are widely used to analyze genotype–phenotype and environment–phenotype relations. These analysis approaches crucially rely on the properties of phenotype ontologies described here.

**Figure 3:**
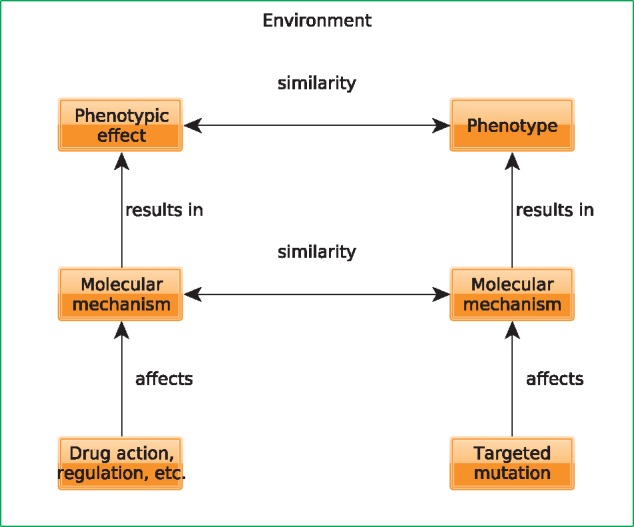
Using phenotype similarity to understand similarity between molecular mechanisms.

One key assumption behind an ontology-based analysis of phenotypes is that a similarity between phenotypes provides information about similarity behind the underlying mechanisms leading to the phenotype (Figure 3). In these types of analyses, phenotypes resulting from a known intervention and mechanism are compared with a set of phenotypes for which the mechanism is not known, and based on phenotype similarity, inferences are made about the unknown mechanism leading to a set of phenotypes. A prime example is the application to disease gene prioritization where known disease-associated genes with their resulting phenotypes (the disease phenotypes) are compared with a set of phenotypes for which the underlying mechanism is not known (e.g. a patient’s phenotypes). High similarity between the sets of phenotypes can then be used to suggest a possible diagnosis for a patient, including the likely mutation underlying the phenotypes [18]. Such an approach can also be used to find new candidate genes when comparing phenotypes resulting from targeted mutagenesis in model organisms with patient or disease phenotypes [138], and several systems for candidate gene prioritization make use of these approaches [78, 99, 130].

For determining phenotype similarity, semantic similarity over phenotype ontologies is the dominant approach [8]. As phenotype ontologies use the entity as main distinguishing feature and include inheritance over parthood axioms asserted for the entity class, semantic similarity will also predominantly exploit these relations. In particular, phenotypes affecting the same entity (e.g. the heart), or a part thereof (e.g. the heart and the left ventricle), will generally be grouped closer together in the phenotype ontologies and therefore be more similar than phenotypes affecting different entities (e.g. heart and liver). Attributes and qualities are then used to further distinguish between phenotypes of the related entities.

Another approach to analyze phenotypes through phenotype ontologies can be applied to direct phenotype statements in an evolutionary context. Through automated reasoning over phenotype ontologies, in particular reasoning over parthood relations, it is possible to complete missing values in character matrices [139], thereby improving their potential use in phylogenetics studies. Furthermore, by generating comparative phenotypes from the direct phenotype statements in character matrices, a pioneering study has identified and confirmed candidate genes that are changed between different species [109]. In this study, direct phenotype statements in fish have been used to generate the comparative phenotypes, and then compared them with mutant zebrafish phenotypes to generate hypotheses about the genetic differences between species.

Furthermore, in comparative statements, PATO contains information about whether the value of an attribute is increased or decreased, and PATO-based phenotype ontologies can therefore also be used to match the ‘directions’ (i.e. increased or decreased values) of phenotypes and find interventions that may be used to treat certain phenotypes. This approach is particularly useful for drug-related work and identifying synergistic drug effects as well as drug effects that are opposite to a disease phenotype (and can be used to find indications for a drug). In particular, to find new potential indications for drugs with a known side-effect profile, it is possible to identify drugs that have an effect with an ‘opposite’ directionality to (some aspects of) the disease phenotype. For this purpose, increased and decreased qualities in the PATO, together with the ‘opposite to’ relation, can be used to identify comparable phenotypes with opposite directionality. Focusing only on increased and decreased values, we can use PATO to distinguish three categories: ‘having an increased value of a quality’, ‘having a decreased value of a quality’ and ‘having an abnormal value of a quality’. If a disease is characterized by an ‘increased value’ of a particular quality (such as glucose concentration in blood), we can aim to find a drug that has a ‘decreased value’ for this quality as a known drug effect. As drugs have multiple effects, and diseases have multiple phenotypes (signs and symptoms), we can automate this search and use a scoring algorithm to estimate the effect of a drug on a disease. For example, if a drug has an effect with an opposite directionality to the disease phenotype, we may assign a 1 to the effect; if the known effect of the drug has no directionality (e.g. ‘abnormal glucose concentration in blood’ as drug effect), or the disease phenotype has no directionality, we may assign a 0 to the effect; and if the drug effect and disease phenotype have the same directionality (e.g. the drug has an ‘increased glucose concentration in blood’ as effect, and the disease has an ‘increased glucose concentration in blood’ as phenotype), we may assign a -1 to the effect. Based on such a scoring algorithm, we can determine a score for a drug–disease pair based on which we may suggest novel drug indications. A similar approach has also been used to identify synergistic drug combinations [140].

## Licensing of ontologies

Ontologies present similar but distinct problems to software when it comes to licensing their reuse. A major criterion for success for many ontologies is that they are adopted and implemented in a variety of settings and form the basis for further development and research [112]. This poses an intrinsic problem, as the original developers need to protect the credit they are due for their original work as it is being reused. A proposed principle [112, 141] of ontology licensing is that they should be made available under an open license if possible but on the condition that the originators are credited when it is reused. This principle is asserted by the OBO Foundry [112], and most OBO Foundry ontologies are licensed with the Creative Commons Attribution (CC-BY), or CC0 (no copyright reserved), license. The CC-BY license requires the originator to be acknowledged when the ontology is reused, and the CC0 license is a donation to the public domain without constraints. There are advantages in using the legal code provided with the Creative Commons licenses, which are carefully designed to have wide coverage in multiple jurisdictions [142].

The strong emphasis on free and open licensing of ontologies is also a response to the restrictive licenses commonly imposed on medical terminologies and ontologies such as UMLS, SNOMED CT or the ICD. Around 40% of the terms within the UMLS have specific licensing restrictions, and some of the most restrictive licenses are associated with SNOMED CT. While a subset of SNOMED CT is available for researchers complying with the terms of the National Library of Medicines UMLS license, the main terminology is maintained by SNOMED International and requires complex licensing depending on the national location and status of the user. This leads to significant problems with integrating SNOMED CT into other ontologies and can restrict the general functionality of databases that use all, of part of, SNOMED CT in their semantics.

## Conclusions

The challenge of describing phenotypes in a consistent and formal manner was among the first addressed by the development of bio-ontologies. From the starting point of relatively simple, pre-composed, hierarchical structures, ontologies and broader semantic frameworks have now been developed to facilitate data retrieval from databases, literature and structured records, data integration and analysis through semantic similarity, over-representation analysis of phenotypes in populations of organisms or molecules, automated reasoning and machine learning. Many of these developments have been facilitated, at least in part, through the use of formal class definitions using the EQ formalism and PATO [16], with significant achievements in the mobilization of model organism data for the study of human disease.

The emergence of Resource Description Framework (RDF) technologies and adoption of linked data approaches such as the EBI RDF platform [143] and the Bio2RDF [144] initiative provide large-scale and powerful resources, which exemplify the breaking of data silos and the mobilization and integration of large and disparate data sets, which would not have been possible without the prior development of the ontologies available today. Specifically, the move toward the development of large integrated phenotype ontologies, such as Uberpheno [145] or PhenomeNET [130], enabled through the systematic application of PATO, has led to common and shared standards for characterizing phenotypes that can be applied across domain and species boundaries and without which data integration would not be successful. Consequently, large integrated phenotype resources such as Monarch [19], to which several model organism databases directly contribute, are now emerging.

In the future, there are still several challenges to overcome for phenotype ontologies. First, not all phenotype ontologies are amenable to the kind of integration and analysis described here. In particular, developing EQ-based axiom patterns often requires manual work by domain experts and cannot always be performed efficiently, although automated systems for identifying phenotypes and decomposing them in EQ statements are becoming available [146]. Yet, even when these patterns are applied to define classes in phenotype ontologies, they are not always applied consistently. Despite the standardization introduced through PATO and other ontologies, there is a degree of interpretation available when choosing entity and quality. For example, whether ‘hearing loss’ is based on an anatomical entity ‘ear’ depends on whether the class also includes sensorineural hearing loss, but this information is not always available or considered and can lead to different formal representations. These incongruities between phenotype ontologies can be observed in particular between different species, and several efforts are underway to improve the standardized formal representation of the axiom patterns in phenotype ontologies [19, 117, 147].

Furthermore, the classification of phenotypes (and diseases) can be greatly improved when more background information about the underlying entity is considered. In particular, anatomical functions are important to assign abnormal processes (i.e. functionings) to the responsible anatomical entity, and this knowledge could improve phenotype ontologies. Furthermore, the axiom patterns used to define phenotypes can be made more precise to help improve the structure of phenotype ontologies. For example, to determine whether ‘abnormal B-cell apoptosis’ (MP:0008781) should be a subclass of ‘abnormal apoptosis’ (MP:0001648), it is necessary to know whether all or only some (B-cell) apoptotic processes are abnormal within the organism; if all apoptotic processes are abnormal, then all B-cell apoptotic processes are abnormal, too, and ‘abnormal apoptosis’ should be considered a subclass of ‘abnormal B-cell apoptosis’. However, currently, these distinctions are not made in phenotype ontologies and cannot be made during annotation.

Phenotypes arise from a genotype within an environment, and, consequently, environmental conditions are necessary for understanding the mechanisms leading to a phenotype. In the future, studies that focus on environmental influences on the phenotype will increase and lead to a deeper understanding of the mechanisms leading to a phenotype [148, 149].

We are now seeing increased use of ontology-based machine learning across the biomedical sciences, particularly with regard to clinical informatics, the stratification of patient populations and discovery of novel relationships between diseases and their pathobiology and genetics. As data in model organism databases grow and phenotype ontologies become increasingly more standardized and interoperable, phenotype data will be increasingly mobilized in support of human disease gene discovery and genetic diagnostics.

Infrastructure funding for ongoing maintenance and development of ontologies and associated tools will be crucial in maintaining the semantic frameworks now being developed. The importance of these activities for the underpinning of our major biomedical resources and databases should not be underestimated.

**Key Points**

- Phenotypes are now increasingly captured by ontologies to facilitate data integration and computational analysis.
- The use of Semantic Web technologies, PATO-based EQ ontology design patterns and standard ontologies such as PATO facilitate interoperability between phenotype ontologies.
- Axioms in phenotype ontologies facilitate many different types of analysis, ranging from identification of molecular mechanisms to understanding evolutionary relationships.

## Notes

**Georgios V. Gkoutos** is a Professor in Clinical Bioinformatics at the University of Birmingham Medical School and the University Hospitals Birmingham NHS Foundation Trust. His main interests lie within the areas of clinical and biomedical informatics, computational biology and translational research aiming at the discovery of molecular origins of human disease and the development of novel disease diagnostic and intervention strategies.

## Notes

**Paul N. Schofield** is a Reader in Biomedical Informatics at the University of Cambridge. His interests are in mouse and human genetics, and building and applying ontologies for phenotypes and disease.

## Notes

**Robert Hoehndorf** is an Assistant Professor in Computer Science at the King Abdullah University of Science and Technology in Thuwal. His research focuses on the applications of ontologies in biology and biomedicine, with a particular emphasis on integrating and analyzing heterogeneous, multimodal data.
